# A new species of *Psyllaephagus* (Hymenoptera: Encyrtidae) from China, parasitoid of *Macrohomotoma sinica* (Hemiptera: Homotomidae) on *Ficus concinna*

**DOI:** 10.3897/BDJ.9.e63253

**Published:** 2021-03-16

**Authors:** Fei Wu, Wenquan Zhen, Zening Yang, Guohao Zu

**Affiliations:** 1 School of Forestry, Northeast Forestry University, Harbin, Heilongjiang, P.R. China, Harbin, China School of Forestry, Northeast Forestry University, Harbin, Heilongjiang, P.R. China Harbin China; 2 Guangxi Key Laboratory of Beibu Gulf Marine Biodiversity Conservation, College of Marine Sciences, Beibu Gulf University, Qinzhou, Guangxi, 535011, P.R. China, Qinzhou, China Guangxi Key Laboratory of Beibu Gulf Marine Biodiversity Conservation, College of Marine Sciences, Beibu Gulf University, Qinzhou, Guangxi, 535011, P.R. China Qinzhou China; 3 College of Horticulture and Landscape, Tianjin Agricultural University, Tianjin, P.R. China, Tianjin, China College of Horticulture and Landscape, Tianjin Agricultural University, Tianjin, P.R. China Tianjin China

**Keywords:** Chalcidoidea, Encyrtinae, Microteryini, psyllid, parasitoid wasp

## Abstract

**Background:**

During the investigation of forest insects in Guilin, Guangxi, encyrtid parasitoid wasps belonging to the genus *Psyllaephagus* were reared from *Macrohomotoma sinica* (Hemiptera: Homotomidae) feeding on *Ficus concinna*.

**New information:**

A new species of *Psyllaephagus* Howard (Hymenoptera: Encyrtidae), *P. guangxiensis* Zu sp. nov., is described from Guangxi, China as a parasitoid of *Macrohomotoma sinica* Yang & Li (Hemiptera: Homotomidae) on *Ficus concinna* (Miq.) Miq. (Urticales: Moraceae).

## Introduction

*Ficus concinna* is an important landscaping tree species, widely distributed in the coastal areas of southern China and has important ornamental and economic value. During the investigation of forest insects in Guilin, Guangxi, *Macrohomotoma sinica* Yang and Li (1984) (Hemiptera: Homotomidae) was found on *Ficus concinna*. Sap feeding by this hemipteran causes visible damage mainly to the tender shoots, resulting in curled leaves and white flocs, which affect the ornamental value of the fig tree. Parasitoid wasps belonging to the genus *Psyllaephagus* Ashmead 1900 (Encyrtidae) were reared from *M. sinica*. The cosmopolitan genus *Psyllaephagus* was established by Ashmead (1900) and currently includes 245 valid species (Noyes 2019), 17 of these species having been recorded from China, including *P. arenariusTrjapitzin 1967*, *P. belanensis* (*Hoffer 1963*), *P. brevicalcaratus* Li 2010, *P. caillardiae* Sugonjaev 1968, *P. colposceniae* Trjapitzin 1969, *P. densiciliatus* Tan and Zhao 1999, *P. elaeagni* Trjapitzin 1967, *P. latiscapusXu et al. 2000b*, *P. longifuniculus* Xu et al. 2000b, *P. longiventra* Li 2010, *P. longiventrisTrjapitzin 1964*, *P. nartshukae* Trjapitzin 1986, *P. nikolskajae* (Trjapitzin 1964), *P. ogazae* Sugonjaev 1968, *P. punctatus* Zhang 2001, *P. stenopsyllae* (Tachikawa 1963) and *P. taiwanus* Xu et al. 2000a (Tan and Zhao 1999, Xu et al. 2000a, Xu et al. 2000b, Ma 2004, Li 2010, Tang et al. 2016, Zhang et al. 2017). Where the biology is known, all species are primary endoparasitoids of the nymphs of Psyllidae (Hemiptera: Psylloidea) (Noyes and Fallahzadeh 2005).

In the present paper, *P. guangxiensis* Zu sp. nov., reared from *M. sinica* Yang & Li (Hemiptera: Homotomidae) on *F. concinna* (Miq.) Miq. (Urticales: Moraceae), is described as new to science.

## Materials and methods

Specimens of the host Homotomidae were collected from *F. concinna* in Guilin City, Xiangshan District, Wanshou Lane and Xicheng Road on 8 August 2018 and 14 July 2020, respectively, then reared in nylon net bags (150 mesh size). When the parasitoid wasps appeared, they were collected and preserved in 99% ethanol. The four reared specimens (2♀♀, 2♂♂) were dissected and mounted on slides according to Noyes (1982). Body lengths were measured with a Leica M205A stereomicroscope, other measurements being taken using a Olympus CX21 optical microscope equipped with a micrometer in the eyepiece. Morphological terminology and abbreviations follow Noyes (2010).

The holotype of the new species is deposited in the 'insect collections' of Tianjin Agricultural University (TJAU), China.

## Taxon treatments

### Psyllaephagus guangxiensis

Zu sp. n.

5B4993FE-0DDC-5BD1-82F1-919B248DF3DF

8BAB0389-7621-4316-A7FD-05E6E207B16F

#### Materials

- **Type status:** Holotype. **Occurrence:** recordedBy: Zu Guo-Hao; individualCount: 1; sex: female; lifeStage: adult; **Taxon:** scientificName: Psyllaephagus guangxiensis; **Location:** country: China; stateProvince: Guangxi; locality: Guilin City, Xiangshan District, Wanshou Lane; verbatimElevation: 150 m; locationRemarks: label transliteration: "Guangxi, Guilin, Wanshou Lane, 02.08.2018, Zu Guohao, Chen Ye, reared from M. sinica (Hemiptera: Homotomidae) on F. concinna"; **Event:** samplingProtocol: reared; eventDate: 29-08-2018; **Record Level:** collectionCode: Insects; basisOfRecord: PreservedSpecimen
- **Type status:** Paratype. **Occurrence:** recordedBy: Zu Guo-Hao; individualCount: 1; sex: female; lifeStage: adult; **Taxon:** scientificName: Psyllaephagus guangxiensis; **Location:** country: China; stateProvince: Guangxi; locality: Guilin City, Xiangshan District, Wanshou Lane; verbatimElevation: 150 m; locationRemarks: label transliteration: "Guangxi, Guilin, Wanshou lane, 02.08.2018, Zu Guohao, Chen Ye, reared from M. sinica (Hemiptera: Homotomidae) on F. concinna"; **Event:** samplingProtocol: reared; eventDate: 29-08-2018; **Record Level:** collectionCode: Insects; basisOfRecord: PreservedSpecimen
- **Type status:** Paratype. **Occurrence:** recordedBy: Zu Guo-Hao; individualCount: 1; sex: male; lifeStage: adult; **Taxon:** scientificName: Psyllaephagus guangxiensis; **Location:** country: China; stateProvince: Guangxi; locality: Guilin City, Xiangshan District, Wanshou Lane; verbatimElevation: 150 m; locationRemarks: label transliteration: "Guangxi, Guilin, Wanshou lane, 02.08.2018, Zu Guohao, Chen Ye, reared from M. sinica (Hemiptera: Homotomidae) on F. concinna"; **Event:** samplingProtocol: reared; eventDate: 29-08-2018; **Record Level:** collectionCode: Insects; basisOfRecord: PreservedSpecimen
- **Type status:** Paratype. **Occurrence:** recordedBy: Zu Guo-Hao; individualCount: 5; sex: male; lifeStage: adult; **Taxon:** scientificName: Psyllaephagus guangxiensis; **Location:** country: China; stateProvince: Guangxi; locality: Guilin City, Xiangshan District, Xicheng Road; verbatimElevation: 150 m; locationRemarks: label transliteration: "Guangxi, Guilin, Xicheng road, 14.07.2020, Zheng Li, reared from M. sinica (Hemiptera: Homotomidae) on F. concinna"; **Event:** samplingProtocol: reared; eventDate: 14-07-2020; **Record Level:** collectionCode: Insects; basisOfRecord: PreservedSpecimen

#### Description

**Female.** Holotype. Length, 2.02 mm (excluding ovipositor). Body generally with metallic lustre; head, mesoscutum, scutellum and axilla with bright green metallic reﬂection; clypeus and metasoma with copper green reﬂection. Antenna black brown, except scape with apical 1/9 yellowish-white, F1, F2 and F3 (partly) ventrally yellow; mandibles brown, pulpi and palpi yellowish-white; tegulae white; legs pale yellow, except hind coxa wih a large brown spot dorsally; wings hyaline; ovipositor apically paler.

**Figure 1.**
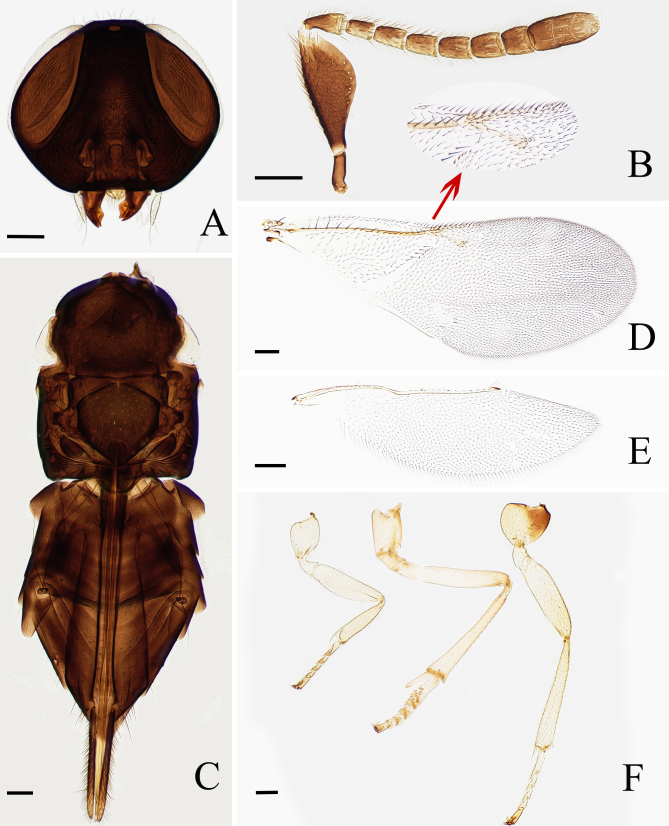
*Psyllaephagus guangxiensis* sp. nov. (female, Holotype): A. head; B. antenna; C. mesosoma and metasoma; D. fore wing; E. hind wing; F. legs. Scale bars = 100 μm.

Frontovertex (Fig. 1A) 0.28× head width, with distinct piliferous punctures of a thimble-like appearance, sculpture reticulate, more longitudinally elongate on lower parts of face and on genae; ocelli forming an angle of 73°; posterior ocellus closer to eye margin than to occipital margin; antennal torulus with its dorsal margin well above lower margin of eyes. Antennal scape (Fig. 1B) broadened, about 2.65× as long as broad; pedicel 2.1× as long as broad, 1.53× as long as F1; F1 1.67×, F2 1.80×, F3 1.80×, F4 1.47×, F5 1.26×, F6 1.09× as long as broad, respectively; clava shorter than preceding three funicle segments combined; funicle with linear sensillae on all funicular segments. Mandible with one tooth and a broad truncation. Measurements (μm): head height, 470; head width, 580; frontovertex width, 160; OD, 33; POL, 103; OOL, 5; OCL, 44; AOL, 48; eye height, 370; malar space, 130; length (and width): radicle, 81; scape, 265 (100); pedicel, 84 (40); F1, 55 (33); F2, 63 (35); F3, 63 (35); F4, 63 (43); F5, 63 (50); F6, 63 (58); clava, 148 (58).

Mesosoma (Fig. 1C). Mesoscutum and scutellum with fine reticulate sculpture; scutellum 1.07× as long as wide and 0.88× as long as mesoscutum. Fore wing (Fig. 1D) 2.28× as long as wide; linea calva closed by one line of setae posteriorly, uninterrupted; postmarginal vein about as long as stigma vein; hind wing (Fig. 1E) 3.55× as long as broad. Mid-tibial spur (Fig. 1F) 0.25× as long as mid-tibia and shorter than corresponding basitarsus. Measurements (μm): fore wing length, 1425; fore wing width, 625; submarginal vein, 590; marginal vein, 40; postmarginal vein, 103; stigmal vein, 108; hind wing length, 975; hind wing width, 275; MT, 560; mid-tibial spur, 140; mid-basitarsus, 190.

Metasoma longer (1.24×) than mesosoma and with hypopygium reaching to about two-thirds in specimens stored in 99% ethanol; ovipositor 2.14× as long as mid-tibia, distinctly exserted; third valvula about 3.14× as long as mid-tibial spur. Measurements (μm): OL, 1200. [MT, 560]

**Figure 2.**
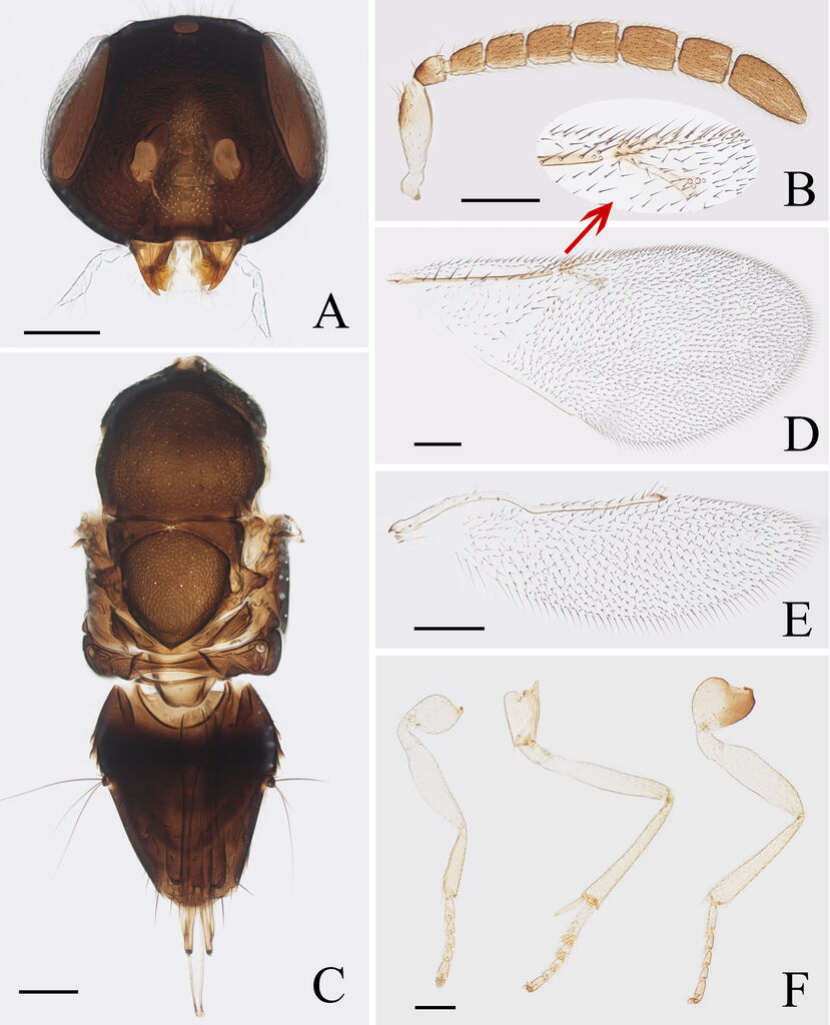
*Psyllaephagus guangxiensis* sp. nov. (male): A. head; B. antenna; C. mesosoma and metasoma; D. fore wing; E. hind wing; F. legs. Scale bars = 100 μm.

**Male.** Length 0.95–1.38 mm. Generally very similar to female in appearance except for colouration of frontovertex, mesoscutum and scutellum with copper green reﬂection, relatively less dense setae in basal cell of fore wing and structure of antennae and genitalia. Head, in frontal view (Fig. 2A), (1.23×) wider than high; frontovertex 0.53× head width, with distinct piliferous punctures of a thimble-like appearance, sculpture polygonal; ocelli forming an angle of 130°; antennal torulus with its dorsal margin well above lower margin of eyes. Antennal scape (Fig. 2B) broadened, about 2.5× as long as broad; all funicle segments subquadrate or slightly longer than wide. Fore wing (Fig. 2D) about 2.17× as long as wide; hind wing (Fig. 2E) 3.30× as long as wide. Mid-tibial spur (Fig. 2F) 0.22× as long as mid-tibia and shorter than corresponding basitarsus. Metasoma (Fig. 2C) shorter than mesosoma; aedeagus about 1.52× as long as mid-tibia.

**Variation.** Very little morphological variation has been found in material included in the type series.

#### Diagnosis

**Female**. Length, 2.02–2.50 mm. Body stout, dark brown, head, mesoscutum, scutellum and axilla with bright green metallic reﬂection; tegulae white; legs pale yellow, except hind coxa partly brown; frontovertex slightly more than a quarter of head width, with distinct piliferous punctures of a thimble-like appearance; ocelli forming an acute triangle; scape broadened, about 2.7× as long as broad; F1 shorter than pedicel; fore wing hyaline, about 2.3× as long as wide; ovipositor distinctly exserted, 2.14× as long as mid-tibia.

**Male** (Length 0.95–1.38 mm). Frontovertex, mesoscutum and scutellum with copper green reﬂection; frontovertex 0.53× head width; scape about 2.5× as long as broad; fore wing about 2.2× as long as broad; aedeagus about 1.5× as long as mid-tibia.

#### Etymology

The specific name refers to the collecting location of the type series. Noun in apposition.

#### Distribution

China (Guangxi).

#### Biology

Parasitoid of *M. sinica* Yang & Li (Hemiptera: Homotomidae) feeding on *F. concinna* (Miq.) Miq. (Urticales: Moraceae).

#### Taxon discussion

According to the keys in Tang et al. (2016) (China), Singh (1996) (India), Trjapitzin (1981) (Palaearctic), Prinsloo (1981) (Southern Africa) and Riek (1962) (Australia), *P. guangxiensis* is similar to *P. macrohomotoma* Singh and Agarwal and *P. bruchus* Riek, which all have a long ovipositor, but *P. guangxiensis* can be distinguished by the broader scape, about 2.7× as long as broad (about 4× in *bruchus*), shorter F1, 0.65× as long as pedicel (longer than pedicel in *macrohomotoma* when compared to figure 20C of Singh and Agarwal (1993), broader fore wing, 2.28× as long as wide (2.46× in *macrohomotoma*) and narrow hind wing, 3.55× as long as broad (3.16× in *macrohomotoma*). The new species is also morphologically similar to *P. elaeagni* Trjapitzin and *P. caillardiae* Sugonjaev. However, it differs from *P. elaeagni* as follows: ovipositor distinctly exserted (not exserted in *elaeagni*), scape 2.7× (5.6× in *elaeagni*), mid-coxa yellow (dark brown in *elaeagni*), tegula completely white (dark brown apically in *elaeagni*); from *P. caillardiae*: scape 2.7× as long as broad (3.8× in *caillardiae*), postmarginal vein about equal to stigmal vein (distinctly shorter than stigmal vein in *caillardiae*).

## Identification Keys

### Key to Chinese species of *Psyllaephagus* (females) [modified from Tang et al. (2016)]

**Table:** 

1	All coxae darkened	2
–	At least one pair of coxae not darkened	9
2	All funicle segments longer than broad	*P. longifuniculus*
–	At least one segment of funicle broader than long or quadrate	3
3	F1-F5 a little longer than broad; F6 quadrate	4
–	F1-F5 not longer than broad; F6 broader than long	6
4	Postmarginal vein absent; all femora darkened	*P. ogazae*
–	Postmarginal vein present; only hind femora darkened	5
5	Scape about as long as the first 4 funicle segments combined	*P. stenopsyllae*
–	Scape longer than the first 4 funicle segments combined	*P. brevicalcaratus*
6	All femora and tibiae at least partly darkened	*P. nartshukae*
–	Only hind femora darkened	7
7	All funicle segments broader than long	*P. nikolskajae*
–	At least one segment of funicle longer than broad	8
8	F1 and F2 slightly longer than broad; F3-F5 subquadrate; F6 broader than long	*P. belanensis*
–	F1 slightly longer than broad; F2-F5 quadrate; F6 slightly broader than long	*P. longiventra*
9	Ocelli forming an obtuse triangle	*P. punctatus*
–	Ocelli forming a right or acute triangle	10
10	Ocelli forming an acute triangle	11
–	Ocelli forming a right triangle	13
11	Tegulae completely white to pale yellow	*P. guangxiensis* **sp. nov.**
–	Tegulae at least partly dark brown	12
12	Tegulae pale yellow for basal 1/2, other wise dark brown; F1-F5 slightly longer than broad, F6 quadrate	*P. elaeagni*
–	Tegulae pale yellow for basal 3/4, other wise dark brown; all funicle segments broader than long	*P. colposceniae*
13	All funicle segments longer than broad	*P. densiciliatus*
–	Not all funicle segments longer than broad	14
14	F1 quadrate; F2–F5 broader than long; F6 subquadrate	*P. taiwanus*
–	F1–F4 longer than broad	15
15	Gaster nearly twice as long as thorax	*P. longiventris*
–	Gaster at most a little longer than thorax	16
16	Tegulae pale yellow for basal 1/2, otherwise dark brown; scape about 2.4× longer than broad	*P. latiscapus*
–	Tegulae pale yellow; scape about 4× longer than broad	*P. caillardiae*

## Supplementary Material

XML Treatment for Psyllaephagus guangxiensis
